# Acute Topiramate Toxicity in a Five-Year-Old Child

**DOI:** 10.7759/cureus.13747

**Published:** 2021-03-07

**Authors:** Mohammad Baidoun, Mohamed Elgendy

**Affiliations:** 1 Pediatrics, Western Michigan University Homer Stryker M.D. School of Medicine, Kalamazoo, USA

**Keywords:** toxicology, inpatient pediatrics, critical care, topiramate, acute encephalopathy

## Abstract

Topiramate (TOPAMAX®) is an anti-epileptic drug for which acute toxicity is infrequently reported.

We present the case report of a five-year-old, otherwise healthy boy who presented to the emergency department (ED) for symptoms of acute encephalopathy. He was lethargic, having slurred speech, hallucinating, intermittently agitated, and had multiple episodes of urinating on himself. Computed tomography (CT) of the head, lumbar puncture, electroencephalography, and magnetic resonance imaging (MRI) were all normal. The urine drug screen was also negative. Two days after admission, a saliva toxicology screen was significant for a topiramate level of 3487.8 ng/ml, which he was not taking and which his mother admitted taking for weight loss. The patient was observed for two days, over which time his symptoms completely resolved, and he was back to baseline.

The following is the take-away for physicians: Careful history-taking should bedone to identify potential drug exposures in children presenting with acute encephalopathy. Especially, given the emerging off-label use of drugs, like in this case, topiramate, which was used by the mother for weight loss. We postulated a possible idiosyncratic reaction vs true drug toxicity, which correlates with findings in a previous case reportout of Boston Children’s Hospital by Taub et al.; and in this case, serum level was about one-third the reported level in this case report^. ^The patient presented with comparable symptoms and time to recovery.

## Introduction

Topiramate is a sulfamate-substituted monosaccharide marketed under the proprietary name TOPAMAX®. The United States Food and Drug Administration (FDA) approved topiramate as initial monotherapy in patients 10 years of age and older with partial-onset or primary generalized tonic-clonic seizures, as adjunctive therapy for adults and pediatric patients ages two to 16 years with partial onset seizures or primary generalized tonic-clonic seizures, and in patients two years of age and older with seizures associated with Lennox-Gastaut syndrome. It is also approved for use in the prophylaxis of migraine headaches.

Topiramate is also used off-label (but is not currently FDA-approved) as a treatment for metabolic disorders, including diabetes mellitus and obesity; eating disorders, such as binge eating disorder and bulimia nervosa [1]; other impulse control disorders, such as pathological gambling; and substance abuse disorders, including alcoholism, nicotine dependence and cocaine abuse [2], cluster headaches [3], essential tremor [4], acute mania [5], and Tourette’s syndrome [6].

The recommended daily dose for topiramate monotherapy in adults and children 10 years of age and older is 400 mg/day. As adjunctive therapy in adults with partial seizures, the dose is 200-400 mg/day while as adjunctive treatment in adults with primary generalized tonic-clonic seizures, the dose is 400 mg/day. As adjunctive therapy for patients two to 16 years-old with partial seizures, primary generalized tonic-clonic seizures, or seizures associated with Lennox-Gastaut syndrome, the dose is approximately 5-9 mg/kg/day. Lastly, the dose for prophylaxis of migraine headache is 100 mg/day.

Neurological and psychiatric abnormalities are both reported in patients using topiramate, usually in the context of chronic dosing. We describe a five-year-old boy, not previously treated with topiramate, who developed acute neurological symptoms after what appeared to be a single episode of topiramate ingestion.

## Case presentation

An otherwise healthy, five-year-old boy presented to the emergency department (ED) with altered mental status, agitation, and visual hallucinations. His mother reported that her son was acting strangely after she came back home at night from a short 30-minute trip. He was in his normal state of good health when he returned home from school earlier that day.

The mother reported that he was combative, agitated, and not responding to her. On presentation to the ED, vital signs were within normal limits for his age: pulse, 69/minute; blood pressure, 112/55 mmHg; respiratory rate, 18/min; temperature, 97.8° Fahrenheit, and oxygen saturation (SpO2) 100% on room air. Examination of the head, eyes, ears, nose, and throat was unremarkable. The neck was supple. The cardiac, lung, abdominal, and skin examinations were all unremarkable. Neurological examination revealed a disoriented male who was intermittently agitated; reflexes and tone were normal.

**Figure 1:**
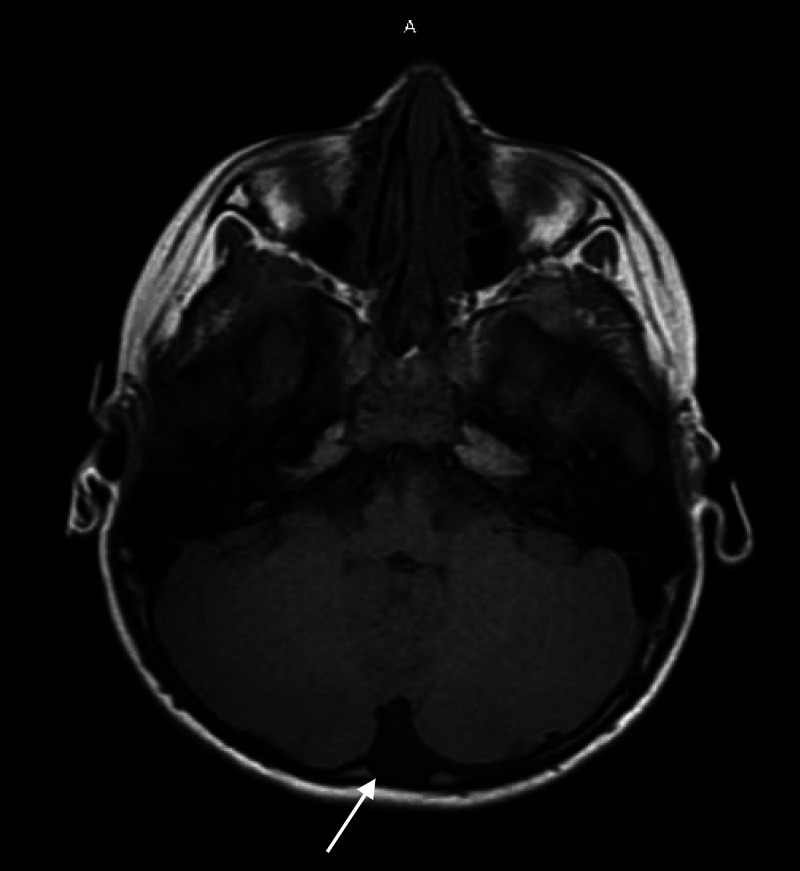
MRI showed a small arachnoid cyst in the left posterior fossa of the left cerebellar hemisphere

The differential diagnosis included sepsis, metabolic disorders, electrolyte derangements, endocrine disorders, hypertension, hepatic failure, renal failure, and drug intoxication. The complete blood count was normal. Serum electrolytes were as follows: sodium, 141 mmol/L; potassium, 3.8 mmol/L; chloride, 108 mmol/L; bicarbonate, 17 mmol/L (normal, 23-32 mmol/L); blood urea nitrogen, 16 mg/dL; creatinine, 0.44 mg/dL; glucose, 110 mg/dL; and calcium, 9.4 mg/dL (Table 1). Urine toxicological testing was negative for amphetamines, barbiturates, cannabinoids, cocaine, methadone, and opiates. Serum acetaminophen and salicylate were undetectable. Computed tomography (CT) of the brain revealed no structural pathology, and subsequent electroencephalography (EEG) was normal. The patient was admitted for observation and lumbar puncture was performed, given the concern for viral encephalitis/meningitis. The patient was transferred to the pediatric intensive care unit (PICU) for worsening altered mental status (AMS), which resolved in a short period of time, and then was transferred to the general inpatient pediatric floor for further care. MRI and EEG were performed, given concern for seizure activity or intracranial brain pathology. MRI revealed a small arachnoid cyst in the left posterior fossa of the left cerebellar hemisphere (Figure 1). Further investigations included ammonia, thyroid-stimulating hormone (TSH), magnesium, erythrocyte sedimentation rate (ESR), procalcitonin, coronavirus disease 2019 (COVID-19) polymerase chain reaction (PCR) and were all negative. Epstein-Barr virus (EBV) serological tests were also negative. A saliva toxicology screen was initially pursued to evaluate for potential drug ingestion. It was drawn on the pediatric floor and returned, two days later, positive for a serum topiramate level of 3487.8 ng/ml. The patient’s symptoms resolved in 48 h. On further history, it was discovered that his mother used topiramate for weight loss. He was then discharged in stable condition.

**Table 1:** Lab results BUN: blood urea nitrogen; EBV: Epstein-Barr virus; COVID-19: coronavirus disease 2019; TSH: thyroid-stimulating hormone

Na	141 mmol/l	Mg	2.5 mol/l
K	3.8 mmol/l	TSH	1.16 mIU/l
CL	108 mmol/l	Ammonia	39 umol/l
HCo3	17 mmol/l	Procalcitonin	0.05
BUN	16 mg/dl	COVID-19 PCR	negative
Serum Cr	0.44 mg/dl	EBV na	6.1
Glucose	110 mg/dl	EBV vca	>8.0
Ca	9.4 mol/l	EBV ea	0.2
EBV serologic studies	Suggest past infection	EBV vca IGM	<0.2
Urine drug screen	Negative for amphetamines, barbiturates, cannabinoids, cocaine, methadone, and opiates	EBV heterophile AB	0.2
Topiramate saliva	3487.8 ng/ml		

## Discussion

Several cognitive/neuropsychiatric adverse events are associated with topiramate use. These include cognitive-related dysfunction (e.g., confusion, psychomotor slowing, difficulty with concentration or attention, difficulty with memory and speech or language problems, particularly word-finding difficulties); psychiatric or behavioral disturbances (e.g., depression or mood problems); and somnolence or fatigue. These symptoms may be minimized if the drug is introduced gradually.

Our patient demonstrated several symptoms that may represent acute neurological toxicity of topiramate including somnolence, intermittent agitation, altered mental status, and hallucinations. A five-year-old girl reported by researchers at Boston Children’s Hospital [7] was found to have a serum topiramate level of 10.5 mcg/mL, which is triple the dose ingested by the patient in our case and recovered in about the same time. Notably, saliva measurement of topiramate can serve as an alternative to serum level, as suggested in “Topiramate concentration in saliva: an alternative to serum monitoring” [8].

In an adult study, oral administration of 100, 200, 400, 800, and 1200 mg of topiramate resulted in peak plasma concentration values of 1.7, 3.7, 7.7, 18.4, and 28.7 mcg/mL, respectively [9]. This patient’s level, as reported, was within the therapeutic range for a patient taking moderate doses of this medication. It is unclear if the patient’s symptoms represented an idiosyncratic reaction, which correlates with findings in the previous case report by Boston Children’s Hospital, as in our case, the saliva level was about one-third the reported level in their case report, both patients presented with comparable symptoms and time to recovery.

## Conclusions

Clinicians should keep exposure to in-home medications in mind when encountering patients with acute encephalopathy. Careful history-taking should be performed to identify any potential exposure in these cases. Given the emerging off-label use of drugs, including topiramate in this situation, all medications need to be taken into consideration when working up acute neurological changes.
